# Genomic Variations in Probiotic *Lactobacillus plantarum* P-8 in the Human and Rat Gut

**DOI:** 10.3389/fmicb.2018.00893

**Published:** 2018-05-08

**Authors:** Yuqin Song, Qiuwen He, Jiachao Zhang, Jianmin Qiao, Haiyan Xu, Zhi Zhong, Wenyi Zhang, Zhihong Sun, Ruifu Yang, Yujun Cui, Heping Zhang

**Affiliations:** ^1^Key Laboratory of Dairy Biotechnology and Engineering, Ministry of Education, Key Laboratory of Dairy Products Processing, Ministry of Agriculture, Inner Mongolia Agricultural University, Hohhot, China; ^2^State Key Laboratory of Pathogen and Biosecurity, Beijing Institute of Microbiology and Epidemiology, Beijing, China

**Keywords:** probiotics, *L. plantarum* P-8, gut, genomic variation, reductive evolution

## Abstract

The effects of probiotics on host gastrointestinal health have become an area of particular interest in the field of probiotic research. However, the impact of the host intestinal environment on genomic changes in probiotic organisms remains largely unknown. To investigate, *Lactobacillus plantarum* P-8, a well-studied probiotic bacterium, was consumed by healthy human volunteers and rats. Then, the persistence and genomic stability of P-8 in the host gut were surveyed. qPCR results revealed that after the consumption of one dose, P-8 could be detected in the host gastrointestinal tract for 4–5 weeks. By contrast, after 4 successive weeks of consumption, P-8 could be detected for up to 17 weeks after consumption ceased. In total, 92 P-8 derived strains were isolated from fecal samples and their genomes were sequenced and analyzed. Comparative genomic analysis detected 19 SNPs, which showed the characteristics of neutral evolution in the core genome. In nearly half of samples (*n* = 39, 42%), the loss of one to three plasmids was observed. The frequent loss of plasmids indicated reductive evolution in the accessory genome under selection pressure within the gastrointestinal tract. We also observed a 609-bp *23S rRNA* homologous fragment that may have been acquired from other species after intake. Our findings offer insight into the complex reactions of probiotics to the gut environment during survival in the host. The *in vivo* genomic dynamics of *L. plantarum* P-8 observed in this study will aid the commercial development of probiotics with more stable characteristics.

## Introduction

The potential health benefits of probiotics is a subject that has gained increasing attention in a health conscious society. Probiotics are live microorganisms that confer health benefits on the host in a safe and efficacious manner when administered in adequate amounts (FAO/WHO, 2002; Hill et al., 2014). The beneficial effects of probiotic bacteria on human health are now widely accepted, and include the production of antimicrobial substances, suppression of the growth of pathogenic bacteria, and modulation of the host immune system (Rijkers et al., 2010; Gerritsen et al., 2011). Therefore, probiotics have become widely available commercially. *Lactobacillus* *plantarum* is traditionally used as a culture starter in the industrial fermentation of raw materials, such as milk and vegetables; and many *L. plantarum* strains have shown a high survival rate after being ingested (Vries et al., 2006). In recent years, some *L. plantarum* strains, such as ST-III (Wang et al., 2011), WCFS1 (Siezen et al., 2012), and P-8 (Zhang et al., 2015a), have been classed as probiotics because of their beneficial effects, including their high survival capacity in the human gastrointestinal tract (GIT), their anti-hyperlipidemic effects, as well as the modulation of gut flora, which were demonstrated by *in vivo* and *in vitro* experiments (van den Nieuwboer et al., 2016).

*Lactobacillus plantarum* strain P-8 (referred to as P-8 below) was originally isolated from a traditionally fermented dairy product in China (Bao et al., 2011). The whole genome of P-8 consists of a circular 3.03 Mb chromosome and seven plasmids, designated LBPp1 to LBPp7 (Zhang et al., 2015b). P-8 exhibits a number of advantageous probiotic properties, including high acid and bile tolerance, good aggregation and antibacterial activities, as well as good stability upon storage (Bao et al., 2012). Further *in vivo* experiments indicated that P-8 exerts beneficial effects on serum lipid reduction (Bao et al., 2012). Moreover, our previous study demonstrated that P-8 could improve human gastrointestinal health and modulate total bile acid and short-chain fatty acid secretion potentially via modulation of the host gut microbiota (Kwok et al., 2015), meanwhile these beneficial effects are likely to be age-related (Wang et al., 2014).

Genetic diversity can be generated by within-host evolution after the invasion of a pathogen (Didelot et al., 2016), similarly, the genome of probiotic strains may also alter following ingestion due to exposure to chemical and physical stresses (i.e., low pH and high bile salt concentration) in the GIT (Douillard et al., 2013), For example, recent *in vitro* evolutionary experiments found that the genomic integrity of *L. rhamnosus* GG was affected by exposure to bile salts or repetitive shearing stress (Douillard et al., 2016). However, little is known regarding the effect of passage through the host GIT on genome variation of *L. plantarum*. Therefore, in the current study, P-8 was fed to human and animal subjects. We determined the dynamics of fecal P-8 abundance, and detected genomic variations in P-8 strains isolated from fecal samples. Our aim was to infer evolutionary changes in P-8 during passage through the GIT, as well as to evaluate its genetic stability within the host GIT environment.

## Materials and Methods

### Ethics Approval and Consent to Participate

All procedures performed in studies involving human participants were in accordance with the ethical standards of the Ethical Committee of Inner Mongolia Agricultural University (Hohhot, China) and with the 1964 Helsinki declaration and its later amendments or comparable ethical standards. All applicable guidelines for the care and use of animals were followed.

### Experimental Design and Sample Collection

We designed three trial groups in this study (Supplementary Figure S1), which employed a total of 36 healthy human individuals and 50 Sprague–Dawley (SD) rats as experimental subjects. Trial 1 included three young volunteers of 26, 24, and 24 years of age. Trial 2 included 50 SD rats that were purchased from Vital River Laboratory Animal Co. Ltd. (Beijing, China). Trial 3 included 33 volunteers that were further assigned to three groups based on their ages: Y group (*n* = 11, average age 26 years), M group (*n* = 12, average age 51 years) and E group (*n* = 10, average age 76 years). All volunteers were healthy individuals, who were non-smokers, with a body mass index <30 kg/m^2^ and a stable weight (±5 kg). All volunteers were required not to take any probiotic-based products and to maintain a typical northern Chinese diet for 2 weeks prior to P-8 administration and during the whole sampling period. All of the rats were acclimatized for 1 week prior to the experiment. Details of the volunteers are listed in Supplementary Table S1.

For trial 1, each volunteer was given a single dose of 6 × 10^10^ CFU of P-8 on day 0. Feces excreted on days 0–7, 14, 21, 28, 35, and 42 were collected. For trial 2, all 50 rats were fed a single dose of 6 × 10^8^ CFU of P-8 on day 0. Three rats were euthanized and dissected on days 0–7, 14, 21, 28, and 35, respectively. Two types of fecal samples were collected from rats at each time point: the fecal samples collected before the euthanasia, and the intestinal mucosal contents scraped after the euthanasia. Trial 3 lasted for 21 weeks (Supplementary Figure S1). Over the first 4 weeks, each subject was given a single oral daily dose of 6 × 10^10^ CFU of P-8. During weeks 5–21, participants received no probiotics. Fecal samples from each subject were collected on weeks 0, 2, 4, 5, 6, 8, 13, 17, and 21. Notably, the samples taken on day 0 and week 0 were collected before probiotic consumption and were used to confirm the absence of P-8 in the gut of volunteers and rats before the experiments commenced. All fecal samples were stored in individual sterile containers and frozen prior to use. The fecal samples from the first 8 weeks of trial 3 had been used to determine the dynamics of biochemical indicators (Wang et al., 2014) and fecal bacterial structure (Kwok et al., 2015) in previous researches.

Throughout the experiment, a total of 408 fecal samples (trial 1: *n* = 39, trial 2: *n* = 72, trial 3: *n* = 297) were collected. For trials 1 and 2, two strains were isolated from each fecal sample in parallel, except that no P-8 strain could be isolated from the samples from days 21, 28, 35, and 42. More over, only one P-8 strain was successfully isolated from R4-1 (rat 1 at day 4) and no P-8 strain was successfully isolated from R7-3 (rat 3 at day 7) in trial 2. For trial 3, only one strain was isolated from each fecal sample, with the exception of samples taken after weeks 13 and 17 from the M and Y groups, respectively, for which no P-8 strain could be isolated. Thus, in total, 108 P-8 descendants (trial 1: *n* = 48, trial 2: *n* = 45, trial 3: *n* = 15) were isolated and used for whole genome sequencing (Supplementary Figure S1 and Supplementary Table S2). Notably, 11 of the 16 strains isolated from the feces of volunteer C in trial 1 did not appear to be descendants of P-8, since their genome sizes and GC contents were significantly smaller than those of P-8. Furthermore, their ANI (average nucleotide identity) values with P-8 as the reference genome were around 70% (Supplementary Figure S2, see method below), which was lower than the cut-off value (ANI < 95%) indicating the same species. Therefore, all genomes from volunteer C of trial 1 were excluded and only the remaining 92 genomes were included in further comparative genomics analysis.

### DNA Extraction From Feces and Quantitative PCR Amplification

Bacterial genomic DNA was extracted from the fecal samples using the QIAamp DNA Stool Mini-Kit (Qiagen, Hilden, Germany) in combination with the bead-beating method (Tanaka et al., 2009), and was stored at -20°C prior to qPCR detection. The qPCR was performed using an ABI Stepone-Plus detection system (Applied Biosystems, Inc., Carlsbad, CA, United States) and primers: *L. plantarum* P-8 primer F: 5′-ACTAACGGGAGGAGTGAT-3′, *L. plantarum* P-8 primer R: 5′-ATAGTTCTCAAATCGGGAC-3′. The reaction mixture (20 μL) contained 10 mM Tris-HCl (pH 8.3), 50 mM KCl, 1.5 mM MgCl_2_, 200 μM of each dNTP, 500 μg/mL bovine serum albumin (Takara, Dalian, China), a 1:75 000 dilution of SYBR Green I (Takara), 0.4 U Taq DNA polymerase Hot Start version (Takara), 0.2 μM of the specific primers and 2 μL template DNA.

### Isolation of P-8 From Feces and DNA Extraction From Isolated P-8 Colonies

After diluting with PBS, the samples were plated and incubated on vancomycin and cycloheximide containing MRS agar under anaerobic conditions for 48 h. To confirm the identity of the P-8 colonies on MRS agar, colony PCR was performed using the strain-specific primers (also used in qPCR). The confirmed P-8 strains were inoculated into MRS liquid medium at 37°C for 48 h. The cells were harvested by centrifugation at 3000 rpm for 8 min, then, half of the cells were used for total genomic DNA extraction using the QIAamp genomic DNA kit (Qiagen) and the rest were preserved by vacuum freeze-drying.

### Whole-Genome Sequencing and Genome Assembly

Genomic DNA was sequenced by the Shanghai Majorbio Bio-Pharm Technology Corporation using Illumina HiSeq 2000 (Illumina) platform. Raw reads of 101 bp in length with an average insert size of 350 bp were generated. After filtering, the average sequencing depth of the high quality data for each sample was higher than 100-fold. The paired-end reads were *de novo* assembled using SOAPdenovo v2.04 (Luo et al., 2012) according to a previous study (Cui et al., 2013). The genome size of 92 P-8 decedents was 3.15 ± 0.03 Mb, and the average GC content was 44.58 ± 0.04%.

### Calculation of the ANI Values

The ANI is the average identity value calculated from a pair-wise comparison of homologous sequences between two genomes and is frequently used in the definition of species (Chan et al., 2012). In the current study, pair-wise ANI values were calculated using a previously described method (Goris et al., 2007).

### Identification of SNPs

Contigs of each strain were aligned to the genome of P-8 to identify SNPs, using MUMmer 3.0 (Kurtz et al., 2004). Then, the SNPs were filtered according to the following criteria: (a) quality scores > 20 (average base calling error rate < 0.01); (b) covered by >10 paired-end reads; (c) not in repetitive regions. In addition, we retrieved the upstream and downstream 100-bp sequences of each SNP in the final set and designed primers (Supplementary Table S3) for PCR amplification. The Sanger sequencing results of PCR products were used to verify the nucleotide status of SNPs acquired from *in silico* analysis.

### Core/Pan Genome Construction

We compared all 92 assembled genomes acquired in this study, as well as the reference genome of P-8, to identify genomic contents shared by all 93 genomes. First, we mapped the assembled contigs of each strain against the genome sequence of the reference P-8 using BLASTn (Altschul et al., 1990) to delineate shared regions (the core genome), with identity levels ≥ 90% and an e-value < 1e-5. Then, the regions that mapped to the core-genome were excluded, and we obtained a set of redundant strain-specific sequences. Further, pair-wise comparisons of these sequences based on BLAT (Kent, 2002) with an identity level ≥ 90% and a match length ≥ 85% resulted in a set of non-redundant sequences, i.e., the accessory genome.

### Genome Fragments Gain and Loss Analysis

Alignment of the accessory genome with the reference genome of P-8 using BLASTn (Altschul et al., 1990) showed that all of the accessory genome fragments were plasmid-born regions, with the exception of one fragment of 609 bp that was similar to part of the 23S rRNA gene located on the chromosome. To investigate the status of each fragment in each strain, we calculated the read coverage and depth of each fecal isolate against the accessory genome: coverage >80% was considered to be present, while coverage <20% was regarded as absent. Furthermore, the other regions of the plasmids were aligned to the chromosome of P-8 to detect any overlap between plasmids and the P-8 chromosome. In addition, plasmid-specific primers (Supplementary Table S4) were designed using primer-BLAST (Jian et al., 2012) to carry out PCR and agarose gel electrophoresis to confirm partial or complete plasmid loss.

### Functional Annotation

To investigate further how the variations related to the functional properties of the proteins, the amino acid sequences were searched against the COG database (Tatusov et al., 2003) and the KEGG database^1^ (Minoru et al., 2016) using BLASTP with the criteria set as: *e*-value < 1e-5, identity > 40% and length coverage of the gene > 50%.

### Nucleotide Sequence Accession Numbers

The complete map of reference genome P-8 (accession number: NC_021224.2) was downloaded from GenBank database. The genome data of the 92 P-8 strains sequenced in this project have been deposited in the NCBI database under the BioProject ID: PRJNA358857, with accession numbers for each assembly were listed in the Supplementary Table S5.

## Results

### Temporary Colonization of P-8 in the Human and Rat Gut

**FIGURE 1:**
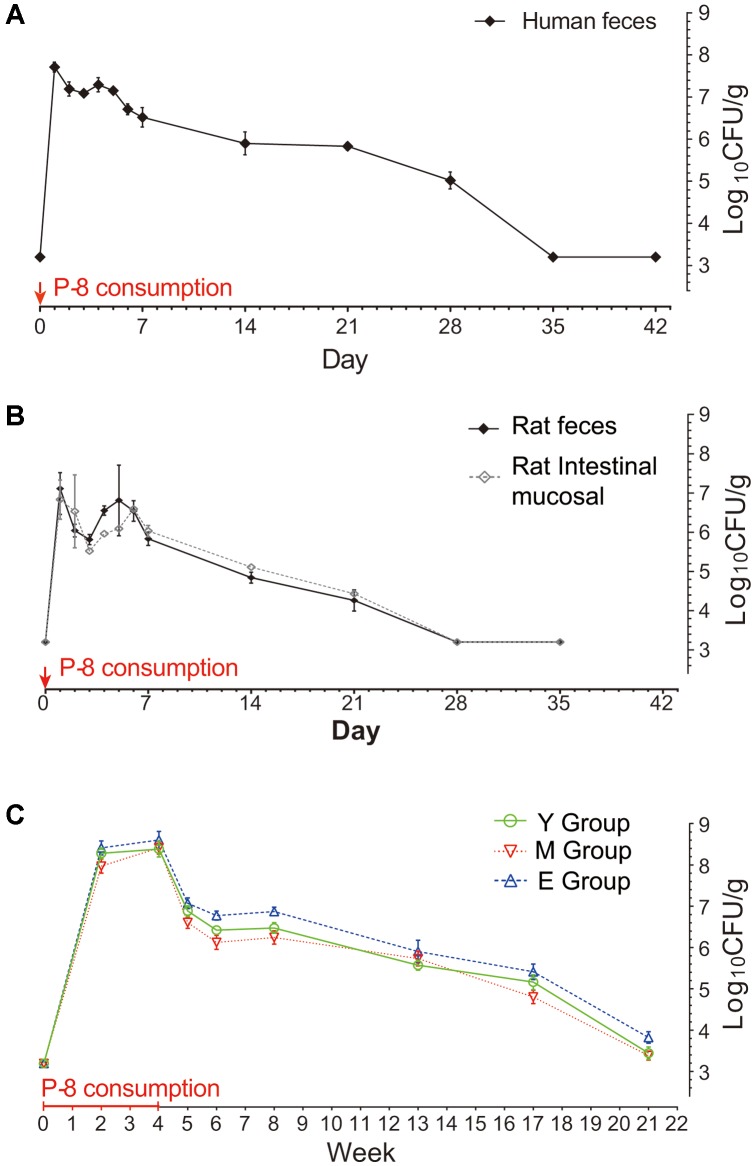
Abundance of fecal P-8 in humans and rats at different time points. **(A)** Trial 1: Human volunteers, 1-day intake of P-8; **(B)** Trial 2: SD (Sprague-Dawley) rats group: black line represents rat feces and gray line represents rat intestinal mucosal scraping, each for 1-day intake of P-8; **(C)** Trial 3: Three groups of human volunteers of different ages. Y group: young, *n* = 11, M group: middle-aged, *n* = 12, E group: elderly, *n* = 10, each volunteer was given P-8 once per day for four successive weeks. The consumption periods are marked with a red line (4 weeks) and arrows (1 day) on the lateral axis. The vertical axis represents the logarithm value for the colony content of fecal samples per gram. Error bars indicate the standard error of the means.

The abundance of P-8 in fecal samples was determined by qPCR targeting of a 262-bp unique sequence of the P-8 chromosome (**Figure 1**). The qPCR results showed that after intake of P-8, the P-8 abundance in feces increased, then dropped to a level lower than the detection limit. Notably, after experiencing a sharp drop from the initial peak, the abundance of P-8 showed a slight increase on day 5 in trial 2 (repeated measures ANOVA, *p* < 0.05) and remained stable on days 3–5 in trial 1 and weeks 6–8 in trial 3 (repeated measures ANOVA, *p* > 0.05), suggesting a brief period of maintenance of P-8 levels in the human gut. In trials 1 and 2, P-8 could only be detected up to 5 weeks after intake, but in trial 3, P-8 could be detected until the 17th week after probiotic consumption had stopped. In trial 3, P-8 abundance peaked around one order of magnitude higher than in trials 1 or 2 (10^8.5^ CFU/g in trial 3, 10^7.6^ CFU/g in trial 1 and 10^7.1^ CFU/g in trial 2), which was measured by calculating the colony content of the fecal sample per gram by qPCR.

### Single Nucleotide Polymorphism (SNP) Variations Among Fecal Isolates

**FIGURE 2:**
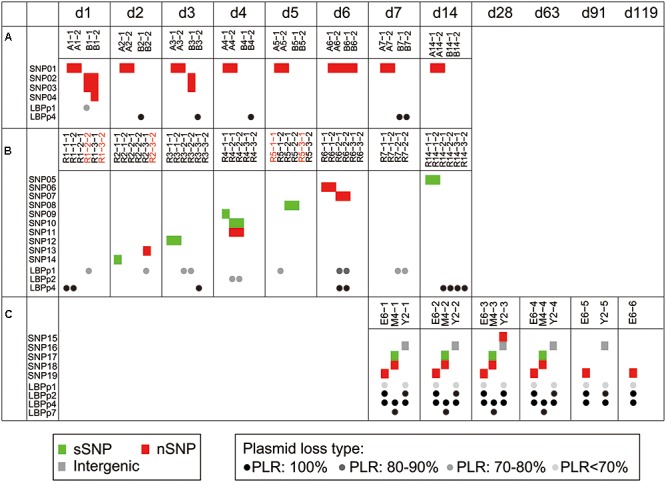
Genome variations identified among 92 fecal-isolated strains. Trial 1 **(A)**, trial 2 **(B)**, and trial 3 **(C)**. Each block (red: non-synonymous SNPs, green: synonymous SNPs, gray: intergenic SNPs) represents an SNP that occurred in the corresponding strain. The dots in black (loss of whole plasmid) and gray (partial plasmid loss) indicate the type of plasmid loss in the corresponding strains. Different levels of gray show different plasmid loss ratios (PLRs). Acquisition events are marked by red strain names. All strains were arranged according to the sampling times. The time points, d1 to d119, represent the time periods after probiotic intake had been ceased in the three trials.

To investigate the genetic stability of the P-8 genome, we surveyed the fecal isolated P-8 strains for single nucleotide polymorphisms. Taking the previously sequenced P-8 genome (Accession No.: NC 021224.2) as a reference (Zhang et al., 2015b), a total of 19 SNPs were identified from the genomes of 92 fecal isolated strains (**Table 1**), of which seven were synonymous, nine were non-synonymous, two were nonsense and one was intergenic. The 18 non-intergenic SNPs separately located to 18 different genes (**Table 1**). Four, ten, and five SNPs were found in strains from trials 1, 2, and 3, respectively (**Figure 2**). However, all SNP loci were trial-specific, or even individual-specific, i.e., the same SNPs were only carried by multiple strains isolated from the same individual, and there was no overlap between different trials or even between different individual subjects. Notably, variations appeared rapidly after P-8 intake in trials 1 and 2 (on the 1st and 2nd days), and 14 SNPs were detected within 2 weeks. However, for each individual human or rat, no more than three SNPs were observed compared with the reference P-8 genome (Supplementary Figure S3), either for strains with short survival times in the gut (trials 1 and 2), or strains with much longer survival periods (trial 3).

**Table 1:** The Information of all detected single nucleotide polymorphisms (SNPs).

SNP_ID	Genome_pos	Mut_type^a^	Mut_base^b^	Mut_pro^c^	Ref_Gene	Product	COG_categories^d^	KO_No.	Pathway_No.^e^
SNP01	1375280	Non-syn	C-T	R61C	LBP RS06445	Membrane protein	–	–	–
SNP02	1059218	Non-syn	G-A	G598S	LBP RS04920	Phosphatidic acid phosphatase	COG0671[I]	K19302	Metabolism[PATH:ko00550]
SNP03	1760063	Nonsense	C-T	Q808X	LBP RS08305	Epimerase	COG1087[M]	K01784	Metabolism[PATH:ko00052,ko00520]
SNP04	2090448	Non-syn	G-T	D358Y	LBP RS10060	Hypothetical protein	–	–	–
SNP05	1441158	syn	G-T	V474V	LBP RS06740	Phosphohydrolase	–	–	–
SNP06	710832	Non-syn	A-G	S160G	LBP RS03240	Bifunctional amino acid aminotransferase/2-hydroxyacid dehydrogenase	COG1052[CHR]	K03778	Metabolism[PATH:ko00620]
SNP07	1859386	Nonsense	G-T	E574X	LBP RS08745	23S rRNA methyltransferase	COG0500[QR]	K00563	–
SNP08	3005584	Syn	A-G	P1509P	LBP RS14195	Hypothetical protein	–	–	–
SNP09	2188536	Syn	T-G	G744G	LBP RS10480	1-deoxy-D-xylulose-5-phosphate synthase	COG1154[HI]	K01662	Metabolism[PATH:ko00730,ko00900]
SNP10	2228459	Syn	C-T	Y81Y	LBP RS10685	Ribonucleotide reductase assembly protein NrdI	COG1780[F]	K03647	–
SNP11	2253167	Non-syn	C-A	P601T	LBP RS10775	Orotidine 5′-phosphate decarboxylase	COG0284[F]	K01591	Metabolism[PATH:ko00240]
SNP12	1929398	Syn	C-T	T2319T	LBP RS09215	Hypothetical protein	–	–	–
SNP13	2570686	Non-syn	G-A	D1942N	LBP RS12200	Cell wall anchor protein	–	–	–
SNP14	2196065	Syn	C-A	V1275V	LBP RS10515	Oligoendopeptidase	COG1164[E]	–	–
SNP15	608137	Non-syn	A-G	T2746A	LBP RS02810	Excinuclease ABC subunit A	COG0178[L]	K03701	Genetic Information Processing [PATH:ko03420]
SNP16	2076616	Intergenic	T-C	–	–	–	–	–	–
SNP17	879878	Syn	T-C	G693G	LBP RS04100	ABC transporter permease	COG0619[P]	K16785	Environmental Information Processing [PATH:ko02010]
SNP18	928900	Non-syn	C-T	T707I	LBP RS04305	Thiol reductant ABC exporter subunit CydD	COG4988[CO]	K16013	Environmental Information Processing [PATH:ko02010]
SNP19	1800964	Non-syn	G-A	M99I	LBP RS08475	GTP-binding protein	COG1217[T]	K06207	–

^a^*The mutation type of the SNPs, non-syn, non-synonymous; syn, synonymous. The mutation type of nucleotides on genome of reference-reisolates. The mutation type and position of amino acids corresponding to SNPs. INFORMATION STORAGE AND PROCESSING: [L]Replication, recombination and repair; METABOLISM: [C]Energy production and conversion;[E]Amino acid transport and metabolism;[F]Nucleotide transport and metabolism;[H]Coenzyme transport and metabolism;[I]Lipid transport and metabolism;[P]Inorganic ion transport and metabolism;[Q]Secondary metabolites biosynthesis, transport and catabolism; CELLULAR PROCESSES AND SIGNALING: [M]Cell wall/membrane/envelope biogenesis;[O]Posttranslational modification, protein turnover, chaperones;[T]Signal transduction mechanisms; POORLY CHARACTERIZED: [R]General function prediction only. Metabolism: [ko00550]Glycan biosynthesis and metabolism; Peptidoglycan biosynthesis; [ko00052]Carbohydrate metabolism; Galactose metabolism; [ko00520]Carbohydrate metabolism; Amino sugar and nucleotide sugar metabolism; [ko00620]Carbohydrate metabolism; Pyruvate metabolism; [ko00730]Metabolism of cofactors and vitamins; Thiamine metabolism; [ko00900]Metabolism of terpenoids and polyketides; Terpenoid backbone biosynthesis; [ko00240]Nucleotide metabolism; Pyrimidine metabolism. Genetic Information Processing: [ko03420] Replication and repair; Nucleotide excision repair. Environmental Information Processing: [ko02010] Membrane transport; ABC transporters*.

### The Loss of Plasmids Is Common in P-8 Fecal Isolates

By comparing the assemblies with the common shared sequences (core-genome) of the 92 fecal isolates and the reference genome, we identified 71 strain-specific sequences that were absent in at least one assembly (accessory-genome, see section “Materials and Methods”), with a total length of 0.31-Mb. We found that almost all accessory genome fragments (70 of the 71 sequences) located on four of the seven plasmids of P-8 (LBPp1, LBPp2, LBPp4, and LBPp7). Only one accessory fragment of 609 bp was similar to part of the 23S rRNA gene located on the chromosome. After excluding the accessory fragments, all of the remaining portions of plasmids LBPp2, LBPp4, and LBPp7 were homologous to regions on the chromosome. Therefore, it can be inferred that these three plasmids may be lost entirely in some fecal P-8 strains. By PCR amplification using plasmid-specific primers (Supplementary Table S4), we confirmed the entire loss of these plasmids in 29 strains (Supplementary Figure S4). LBPp4 was deleted in five strains from trial 1, nine strains from trial 2 and all strains from trial 3. The deletion of LBPp7 only occurred in four strains of trial 3, while LBPp2 was deleted in 11 strains of trial 3.

**FIGURE 3:**
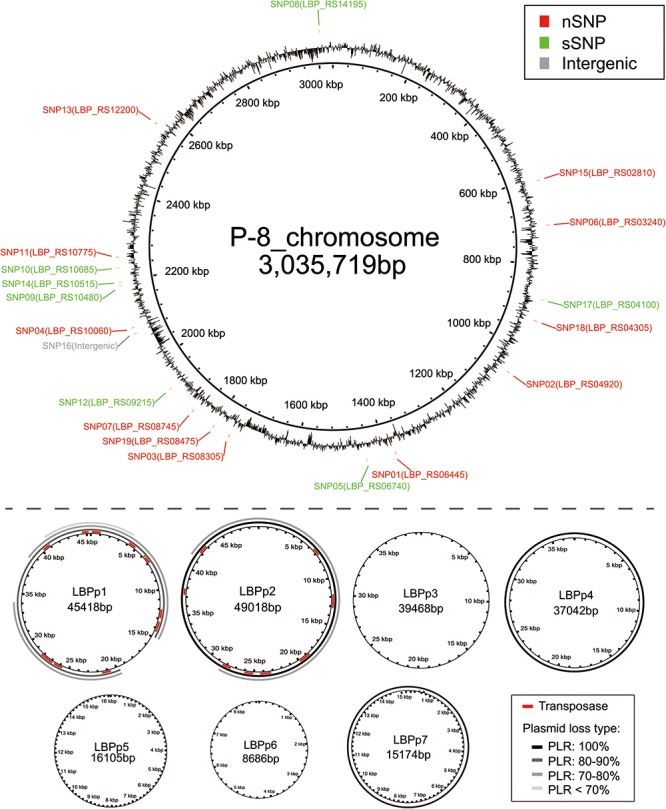
The location of genome variations on the reference P-8 genome. The P-8 chromosome, showing the distribution of the SNPs (red: non-synonymous SNPs, green: synonymous SNPs, gray: intergenic SNPs), is shown above the dotted line. The seven plasmids of P-8 are shown below the dotted line. For LBPp1 and LBPp2, the red regions show the positions of the transposases. For plasmids LBPp1, LBPp2, LBPp4, and LBPp7, each ring, from inside to outside, represents a loss type. The black rings (entirely loss) and gray arcs (partially loss) indicate the plasmid loss regions, and the gray levels show the different plasmid loss ratios (PLRs).

Besides the complete loss of one or more plasmids, the partial loss of LBPp1 and LBPp2 was detected in 23 strains. Here, we defined a term, the plasmid loss ratio (PLR), to indicate the ratio of the length of the missing region to that of the whole plasmid. Based on the PLR, we classified the plasmid loss status into four plasmid loss types (PLR: 100%, PLR: 80–90%, PLR: 70–80%, and PLR <70%, as shown in **Figure 3**). There were three different types of deletions of LBPp1, and two types of LBPp2 deletions. The loss of LBPp1 was detected in all three trials (**Figure 2**). A PLR of 70–80% of LBPp1 occurred in one strain of trial 1 and seven strains of trial 2, and a PLR of 80–90% was detected in two strains of trial 2. A PLR <70% (an 8.47-kb region) was observed in 11 strains of trial 3. The partial deletion of LBPp2 was detected in only two strains of trial 2 (PLR of 70–80%). In general, it seemed that plasmid loss was a common phenomenon in all three trials.

### One Genome Fragment Might Be Acquired by Fecal Isolates

There were five copies of the 23S rRNA gene in the genome of P-8, which were 2923 bp in length and had pair-wise identity values above 99.9%. The 609-bp fragment mentioned above presented in five strains of trial 2 and showed high identity (90%) to the 23S rRNA gene of the reference P-8 from 1215 to 1823 bp. Alignment against the nr database^2^ indicated that this fragment showed a high identity value of 97% with the 23S rRNA genes of DSM20016, the type strain of the species *L. reuteri*. It was reported that *L. reuteri* was one of the dominant species of the intestinal flora of animals (Oh et al., 2010). Therefore, it can be inferred that this fragment might be acquired by P-8 isolates through HGT (horizontal gene transfer) in the gut.

### Functional Annotation of the Variations

The 18 SNP-carrying genes were randomly distributed across the chromosome (**Figure 3**). The annotation results indicated that of the 18 genes, 12 (66.7%) could be classified into 12 COG functional categories (**Table 1**), among which metabolism-related genes were the most abundant category. Furthermore, 11 genes could be classified into nine KEGG pathways. Most of the SNP encoded proteins in trials 1 and 2 were involved in metabolic pathways, including those for carbohydrate, nucleotides, cofactors and vitamins, terpenoids and polyketides, as well as glycan biosynthesis and metabolism (**Table 1**). The SNP encoded proteins in trial 3 were found to function in genetic information processing (ko03420: nucleotide excision repair) and environmental information processing (ko02010: ABC transporters).

**FIGURE 4:**
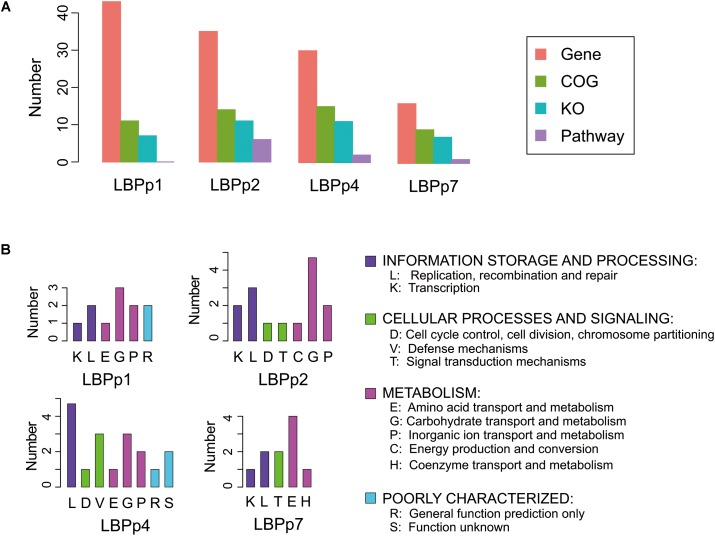
Functional annotation information of the four lost plasmids. **(A)** Barplot of the number of genes (orange), COGs (green), KOs (blue), and pathways (purple) of the four plasmids. The KOs were the enzymes predicted by homology analysis based on the KEGG database, and the pathways refer to the metabolic pathways that the KOs may participate in. **(B)** The categories of COGs in each plasmid.

The biological functions of the four lost plasmids were largely unknown. Only 9 to 15 proteins of the four plasmids were classified into known functional categories (**Figure 4A**). The functional categories involved in information storage and processing (K/L) and metabolism (E/G/P/C/H) were detected in all plasmids (**Figure 4B**). There were three genes associated with the functions of information storage and processing in LBPp1, these included genes encoding the transposase (LBP RS14410), resolvase (LBP RS14420) and metal-dependent transcriptional regulator (LBP RS14435) (Supplementary Table S6). LBPp2 and LBPp4 both possessed two more genes relating to these functions than LBPp1, which encoded the transposase and topoisomerase, but may be superfluous genes for P-8. LBPp7 also possessed three genes belonging to the K/L categories, one of which was a transposase. As for the numbers of genes in categories involved in metabolism, LBPp2, the most abundant, possessed eight metabolism-related genes, five of which encoded galactosidases. This was followed by LBPp1 and LBPp4, each with six metabolism-associated genes, most of which encoded permeases, sugar transporters and dihydroxyacetone kinases involved in carbohydrate transport and metabolism. Five genes belonging to LBPp7 had functions in metabolism, and four of these encoded components of the ABC-type amino acid transport system. Furthermore, genes belonging to functional categories involved in cellular processes and signaling (D/V/T) were detected in LBPp2, LBPp4, and LBPp7. Among them, LBPp2 possessed two genes encoding a response regulator (LBP RS14710) and a replication-associated protein RepB (LBP RS14755). LBPp4 possessed three genes that encoded a complete type I restriction–modification system. Moreover, one of the two T category genes of LBPp7 encoded a type II toxin–antitoxin system PemK/MazF family toxin (LBP RS15390).

## Discussion and Conclusion

The ability to survive in the host GIT is an important feature of probiotics. Previous studies have shown that many *L. plantarum* strains have a high survival rate in the human GIT following ingestion (Vries et al., 2006). The probiotic *L. plantarum* strain, P-8, is characterized by its high tolerance in acidic and bile-containing environments, and its strong *in vitro* antibacterial activity (Bao et al., 2012). In this study, experiments involving the oral consumption of P-8 were performed on humans and rats. Fecal quantification of ingested probiotic strains can be used to reflect the bacterial cell death rate (mainly in the upper GIT), and the subsequent replication of surviving cells (Derrien and van Hylckama Vlieg, 2015). Thus, along the course of the experiment, the dynamics of fecal P-8 abundance were monitored by qPCR. The levels of fecal P-8 abundance were high during the consumption period, decreasing as soon as consumption ended. This was consistent with previous reports on *L. plantarum* WCFS1 in human feeding trials (Vesa et al., 2000) and oral administration experiments with other lactobacilli (Saxelin et al., 1995; Jacobsen et al., 1999; Hütt et al., 2011). However, a slight increase or stable levels of P-8 in fecal samples were observed after several days both in human and rat trials, indicating that P-8 might temporally propagate in the human and rat GIT. Moreover, in most cases, after ceasing consumption, the ingested strains could only be detected for another few days or up to 1 week (Firmesse et al., 2008; Fujimoto et al., 2008). Our results indicated that after a 1-day intake of P-8, the probiotic could be detected up until the 4th or 5th week. With continuous consumption of P-8 over a 4-week period, the bacteria persisted in the host gut longer and could still be detected up until the 17th week after consumption had been ceased. Thus, our results suggested that it may be important to maintain continuous consumption to ensure that the abundance of probiotics in the gut remains at an adequate level to exert their beneficial properties.

The species *L. plantarum* is a ubiquitous microorganism that can be found in many different ecological niches including food stuffs such as vegetables, meat, fish, and dairy products, as well as the GIT (Siezen et al., 2010). Previous studies revealed high genetic diversity and niche-specific lineages present within the *L. plantarum* species, suggesting that adaptive variations occur within the *L. plantarum* genome in response to diverse environmental niches (Molenaar et al., 2005; Siezen et al., 2010; Xu et al., 2015). Furthermore, a comparative genome analysis of *L. rhamnosus* GG discovered that deletion of the genomic island LGGISL1,2 was linked to probiotic functionality and the genetic instability of *L. rhamnosus* GG (Sybesma et al., 2013). Therefore, in the current study, it was important to investigate the variability of P-8, a probiotic of dairy origin, during passage through the GIT. The probiotic P-8 contains a circular chromosome and seven plasmids designated LBPp1 to LBPp7 (Zhang et al., 2015b). Our results showed that the majority of P-8 strains isolated from fecal samples in all three trials had lost one to three plasmids, indicating a possible trend toward genome reduction within the host. This was consistent with the evolutionary process undergone by some pathogens (Moran, 2002), such as *Yersinia pestis* (Wren, 2003) and *Rickettsia* (Merhej and Raoult, 2011), from their environmental ancestors. Host-adapted pathogens can obtain many intermediate metabolic products from the host, thereby allowing for reductive evolution in the corresponding biosynthetic pathways and genes, such as the pathways for carbohydrate, amino acid and nucleotide biosynthesis and the genes involved in energy metabolism (Moran, 2002). It was also reported that gene decay or loss of superfluous genes in the lactobacilli genome was a possible result of the adaptation from nutritionally variable environments to the relatively constant and nutrient-rich dairy niche (van de Guchte et al., 2006; Cai et al., 2009), thereby reducing the energy required for non-essential gene expression. Obviously, the GIT contains lots of nutrients and provides another kind of nutrient-rich niche environment for strains such as P-8. Thus, genes involved in carbohydrate and amino acid transport and metabolism might become superfluous functionally in such an environment. Plasmids LBPp1, LBPp2, LBPp4, and LBPp7 all encoded these types of genes, especially LBPp2 that possesses five galactosidase genes that play an important role in dairy fermentation. This might be one possible explanation for the observed deletion of these plasmids. The probiotic P-8 was isolated from a natural fermented dairy product, which usually contains a high number of lactic acid bacteria and occasionally phage from the natural environment (Garneau and Moineau, 2011). We detected genes from type I restriction–modification systems and type II toxin–antitoxin systems within LBPp4 and LBPp7. Both of these systems are responsible for the defense against invasion of foreign DNA, i.e., phage (Hazan and Engelberg-Kulka, 2004; Labrie et al., 2010), but are likely useless for survival in the animal gut. Furthermore, it should be noted that the GIT is also an extremely harsh chemical and physical environment with a low pH and a high bile salt concentration, which would exert a selective pressure on P-8 strains. The majority of P-8 strains would not survive passage through the GIT, leading to a rapid decrease in population size. Then, the loss of the plasmids might be a stress response of P-8 strains to conserve energy and nutrients and thereby aid survival and adaptation to the GIT environment.

A 609-bp fragment, closely related to the *23S rRNA* gene sequence of *L. reuteri*, was found to be acquired by five fecal P-8 strains in trial 2 in this study. Ribosomal RNAs are encoded by highly conserved genes that play an important role in the catalytic activity of protein synthesis. The *23S rRNA* plays a key role in the peptide elongation phase (Nissen, 2000). P-8 strains experience extensive changes in their environment when entering the GIT, and the synthesis of many proteins may be induced in response to this new environment. Thus, the acquisition of the *23S rRNA* sequence might be a result of this change in environment.

It is reasonable to assume that on exposure to the harsh and complex environment of the gut, probiotics would be subjected to strong selection pressure and correspondingly adaptive changes would be observed. Indeed, exposure of the probiotic *L. plantarum* WCFS1 to the murine intestine revealed 25 SNPs in 13 adapted derivatives, suggesting that adaptive changes occur during the persistence of probiotics in the GIT of animals (Veen et al., 2013). However, the distribution pattern and functional characteristics of SNPs identified in this study seemed random, and did not reveal any evidence of evolution acting under selection. First, we identified 19 SNPs in total, of which, 18 non-intergenic SNPs were distributed among 18 different genes rather than clustering in one or several genes that would be indicative of positive selection. Second, functional annotation results showed that the SNP-containing genes of different trials did not group in the same known metabolic pathways, suggesting the lack of selection at the pathway level. These findings suggested that fecal P-8 strains evolved without an obvious selective pressure. Finally, it had been reported that in the absence of any selective pressure, the spontaneous mutation rate was estimated to be 0.0033 changes per generation, regardless of genome size (Drake, 1991). In our study, as fecal P-8 strains persisted in the human gut for 17 weeks in trial 3, and there were one or two SNPs in each strain, the mutation rate of P-8 was estimated to be no larger than 0.0029 changes per generation, as calculated using a previously described method (Drake, 1991). This was approximately equivalent to the spontaneous mutation rate indicating that P-8 was stable at the core-genome level and evolved at a relatively low rate in the GIT. Taken together, our findings indicated neutral evolution of the core genome of the probiotic P-8 during passage through the GIT. Notably, mutations occurred rapidly after intake in trials 1 and 2, with 14 substitutions observed within a week and SNPs appearing even on the 1st day of intake in trial 1 (**Figure 2**). One possible explanation would be that population diversity had already presented in the probiotic tablets/culture used to feed the experimental subjects. After consumption and a sharp reduction in the population size, P-8 experienced a bottleneck and consequently variants were randomly retained in the GIT of each individual. However, this hypothesis needs to be verified by meta-genome or single-cell genome sequencing of the probiotic tablets/culture before intake, which will be addressed in future studies.

In conclusion, in the current study, we evaluated the survival ability and genomic variations of the probiotic *L. plantarum* P-8 in the host gut. We found that long term successive intake of probiotics significantly extended the survival time of this organism in the host gut. At the single nucleotide substitution level, there was no clear evidence of the presence of a selection signal. However, we observed the frequent loss of plasmids in all three trials, suggesting that the probiotic strain experienced reductive evolution in the GIT environment. Current analyses were based on short-read sequencing technology, therefore some types of variation, such as copy number variations of repeat elements and genome rearrangements, may have been difficult to detect. In future studies, longer-term laboratory experiments, accompanied by meta-genomics sequencing and third generation long-read sequencing technology, will provide further insight into the population diversity and microevolution of P-8 in the GIT.

## Author Contributions

HZ and ZS conceived and designed the experiments. QH, JZ, JQ, HX, ZZ, and WZ performed the experiments. YS, ZS, and YC analyzed the data. YS and YC drafted the manuscript. HZ and RY helped to draft the manuscript. All authors read and approved the final manuscript.

## Conflict of Interest Statement

The authors declare that the research was conducted in the absence of any commercial or financial relationships that could be construed as a potential conflict of interest.

## Footnotes

1 http://www.kegg.jp/

2 https://blast.ncbi.nlm.nih.gov/Blast.cgi
