# Supplementary material for: Genomic Variations in Probiotic Lactobacillus plantarum P-8 in the Human and Rat Gut
Source: Front Microbiol. 2018 May 8;9:893. doi: 10.3389/fmicb.2018.00893 (PMC5951974; doi:10.3389/fmicb.2018.00893)
Supplement: Supplementary file 1 [file Data_Sheet_1.docx]

Genomic variations in probiotic *Lactobacillus plantarum* P-8 in the human and rat gut

Yuqin Song^1,☨^, Qiuwen He^1,☨^, Jiachao Zhang^1^, Jianmin Qiao^1^, Haiyan Xu^1^, Zhi Zhong^1^, Wenyi Zhang^1^, Zhihong Sun^1^, Ruifu Yang^2^, Yujun Cui^2,*^, Heping Zhang^1,*^

^1^ Key Laboratory of Dairy Biotechnology and Engineering, Ministry of Education, Key Laboratory of Dairy Products Processing, Ministry of Agriculture, Inner Mongolia Agricultural University, Huhhot 010018, China.

^2^ State Key Laboratory of Pathogen and Biosecurity, Beijing Institute of Microbiology and Epidemiology, Beijing 100071, China.

^☨^ These authors contributed equally to this work.

^*^ Correspondence: hepingdd@vip.sina.com, cuiyujun.new@gmail.com.

**SUPPLEMENTARY FIGURE CAPTIONS**


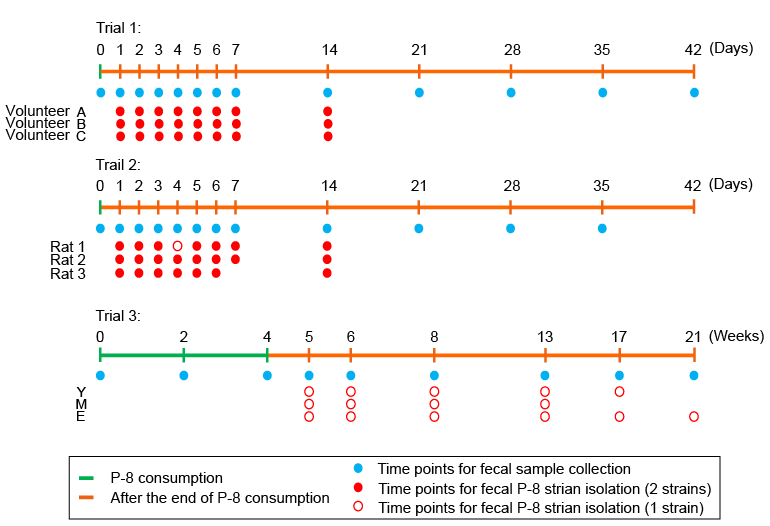


**Figure S1. The experimental design and sample collection.** We defined three trials. Trial 1 and trial 2 lasted for 42 days, while trial 3 for 21 weeks. Green lines indicated the P-8 consumption period (first day of trial 1&2 and first four weeks for trial 3). Orange lines showed the period after the end of P-8 consumption. Blue dots represented the time points for fecal sample collection. Additionally, in trial 2, we also collected intestinal mucosal scraping samples of each rat. Red dots represented the time points at which two suspected fecal P-8 decendents were isolated, while the red circles represented the time points at which only one suspected fecal P-8 decendent was isolated.


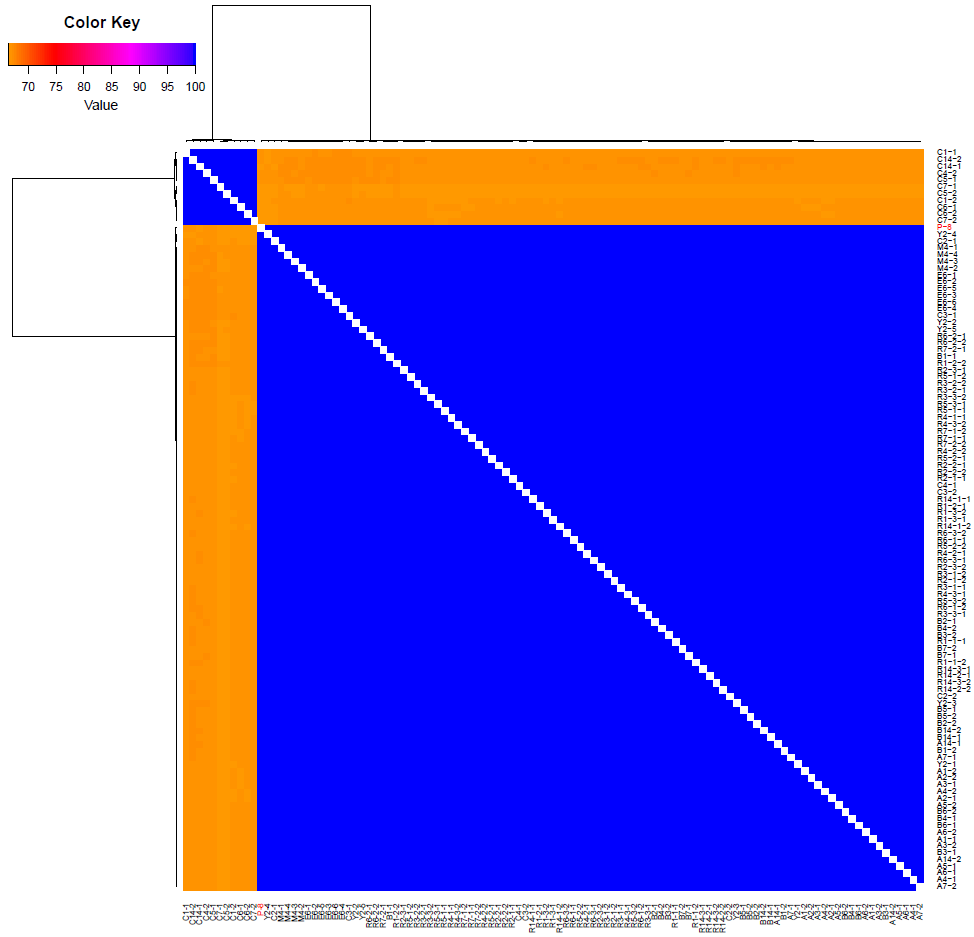


**Figure S2. The heatmap of the pairwise ANI values of 92 genomes together with reference P-8.** The name of reference strain was colored in red.


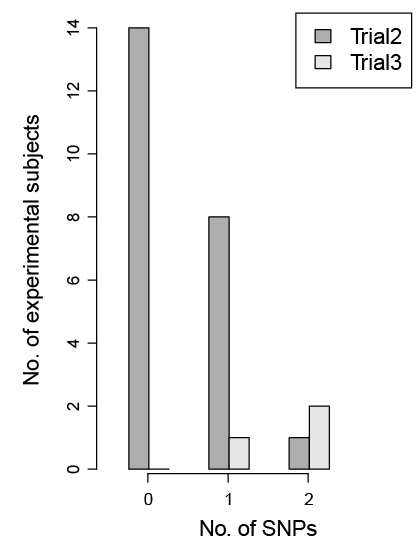


**Figure S3. The histogram based on SNP numbers observed in each individual human or rat in three trials.** The horizontal axis was the number of SNPs detected within each individual experimental subject, and the vertical axis was the number of the experimental subjects carrying corresponding SNPs. Different grey degree presented different trials.


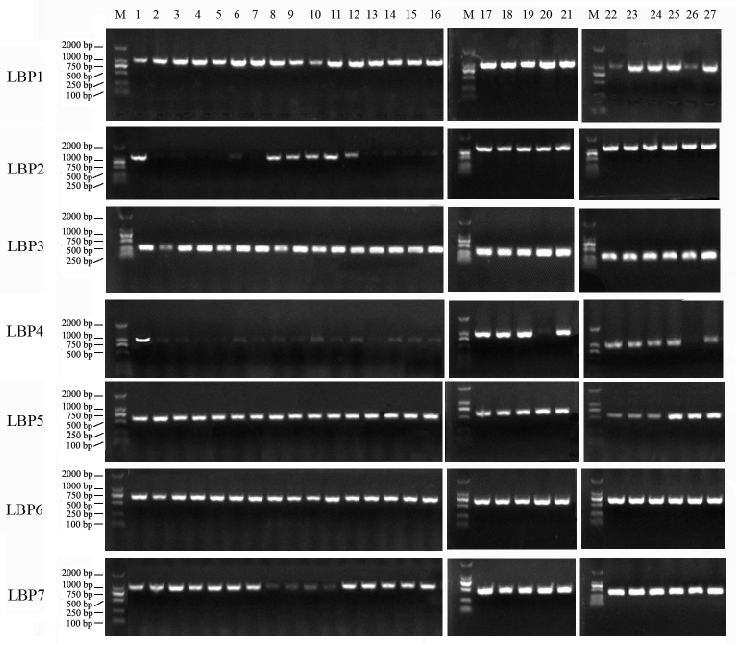


**Figure S4. The agarose gel electrophoresis of partial re-isolates on validation of plasmid loss.** M referred to marker, 1 stood for reference P-8, 2-16 for all 15 strains of trial3, and 17-21 represented 5 strains of trial1, while 22-27 were for 6 strains of trial2.

| Table S1. Information of Volunteers. | | | | | | | |
| --- | --- | --- | --- | --- | --- | --- | --- |
| **Volunteers** | **Gender** | **Age** | **BMI** | **Volunteers** | **Genderr** | **Age** | **BMI** |
| A | Male | 26 | 23.1 | M5 | Male | 52 | 19.6 |
| B | Famele | 24 | 22.7 | M6 | Female | 52 | 25.9 |
| C | Male | 24 | 23.2 | M7 | Male | 50 | 28.7 |
| Y1 | Female | 29 | 19.6 | M8 | Female | 51 | 20.1 |
| Y2 | Male | 25 | 26.5 | M9 | Male | 52 | 21.3 |
| Y3 | Female | 25 | 22.8 | M10 | Female | 53 | 29.3 |
| Y4 | Female | 26 | 21.4 | M11 | Male | 51 | 22.5 |
| Y5 | Female | 27 | 27.2 | M12 | Male | 52 | 24.1 |
| Y6 | Male | 26 | 22.5 | E1 | Male | 71 | 21.5 |
| Y7 | Female | 26 | 23.4 | E2 | Male | 73 | 27.4 |
| Y8 | Male | 26 | 24.6 | E3 | Female | 71 | 26.3 |
| Y9 | Male | 25 | 25.4 | E4 | Male | 75 | 25.8 |
| Y10 | Female | 27 | 26.1 | E5 | Female | 72 | 22.6 |
| Y11 | Male | 26 | 22.9 | E6 | Male | 80 | 19.8 |
| M1 | Female | 48 | 25.7 | E7 | Female | 78 | 22.4 |
| M2 | Male | 50 | 26.4 | E8 | Female | 79 | 26.3 |
| M3 | Female | 51 | 28.4 | E9 | Female | 77 | 25.5 |
| M4 | Female | 49 | 27.5 | E10 | Male | 75 | 20.4 |
| Note: BMI stands for body mass index. | | | | | | | |

| Table S2. Information of reisolatede strains used for whole genome sequencing. | | | |
| --- | --- | --- | --- |
| Samples | Sampling time | Samples | Sampling time |
| A1-1 | Strain1 isolated at day 1 after P-8 consumption of volunteer A | R1-1-1 | Strain1 isolated at day 1 after P-8 consumption of Rat1-1 |
| A1-2 | Strain2 isolated at day 1 after P-8 consumption of volunteer A | R1-1-2 | Strain2 isolated at day 1 after P-8 consumption of Rat1-1 |
| A2-1 | Strain1 isolated at day 2 after P-8 consumption of volunteer A | R1-2-1 | Strain1 isolated at day 1 after P-8 consumption of Rat1-2 |
| A2-2 | Strain2 isolated at day 2 after P-8 consumption of volunteer A | R1-2-2 | Strain2 isolated at day 1 after P-8 consumption of Rat1-2 |
| A3-1 | Strain1 isolated at day 3 after P-8 consumption of volunteer A | R1-3-1 | Strain1 isolated at day 1 after P-8 consumption of Rat1-3 |
| A3-2 | Strain2 isolated at day 3 after P-8 consumption of volunteer A | R1-3-2 | Strain2 isolated at day 1 after P-8 consumption of Rat1-3 |
| A4-1 | Strain1 isolated at day 4 after P-8 consumption of volunteer A | R2-1-1 | Strain1 isolated at day 2 after P-8 consumption of Rat2-1 |
| A4-2 | Strain2 isolated at day 4 after P-8 consumption of volunteer A | R2-1-2 | Strain2 isolated at day 2 after P-8 consumption of Rat2-1 |
| A5-1 | Strain1 isolated at day 5 after P-8 consumption of volunteer A | R2-2-1 | Strain1 isolated at day 2 after P-8 consumption of Rat2-2 |
| A5-2 | Strain2 isolated at day 5 after P-8 consumption of volunteer A | R2-2-2 | Strain2 isolated at day 2 after P-8 consumption of Rat2-2 |
| A6-1 | Strain1 isolated at day 6 after P-8 consumption of volunteer A | R2-3-1 | Strain1 isolated at day 2 after P-8 consumption of Rat2-3 |
| A6-2 | Strain2 isolated at day 6 after P-8 consumption of volunteer A | R2-3-2 | Strain2 isolated at day 2 after P-8 consumption of Rat2-3 |
| A7-1 | Strain1 isolated at day 7 after P-8 consumption of volunteer A | R3-1-1 | Strain1 isolated at day 3 after P-8 consumption of Rat3-1 |
| A7-2 | Strain2 isolated at day 7 after P-8 consumption of volunteer A | R3-1-2 | Strain2 isolated at day 3 after P-8 consumption of Rat3-1 |
| A14-1 | Strain1 isolated at day 14 after P-8 consumption of volunteer A | R3-2-1 | Strain1 isolated at day 3 after P-8 consumption of Rat3-2 |
| A14-2 | Strain2 isolated at day 14 after P-8 consumption of volunteer A | R3-2-2 | Strain2 isolated at day 3 after P-8 consumption of Rat3-2 |
| B1-1 | Strain1 isolated at day 1 after P-8 consumption of volunteer B | R3-3-1 | Strain1 isolated at day 3 after P-8 consumption of Rat3-3 |
| B1-2 | Strain2 isolated at day 1 after P-8 consumption of volunteer B | R3-3-2 | Strain2 isolated at day 3 after P-8 consumption of Rat3-3 |
| B2-1 | Strain1 isolated at day 2 after P-8 consumption of volunteer B | R4-1-1 | Strain1 isolated at day 4 after P-8 consumption of Rat4-1 |
| B2-2 | Strain2 isolated at day 2 after P-8 consumption of volunteer B | R4-2-1 | Strain1 isolated at day 4 after P-8 consumption of Rat4-2 |
| B3-1 | Strain1 isolated at day 3 after P-8 consumption of volunteer B | R4-2-2 | Strain2 isolated at day 4 after P-8 consumption of Rat4-2 |
| B3-2 | Strain2 isolated at day 3 after P-8 consumption of volunteer B | R4-3-1 | Strain1 isolated at day 4 after P-8 consumption of Rat4-3 |
| B4-1 | Strain1 isolated at day 4 after P-8 consumption of volunteer B | R4-3-2 | Strain2 isolated at day 4 after P-8 consumption of Rat4-3 |
| B4-2 | Strain2 isolated at day 4 after P-8 consumption of volunteer B | R5-1-1 | Strain1 isolated at day 5 after P-8 consumption of Rat5-1 |
| B5-1 | Strain1 isolated at day 5 after P-8 consumption of volunteer B | R5-1-2 | Strain2 isolated at day 5 after P-8 consumption of Rat5-1 |
| B5-2 | Strain2 isolated at day 5 after P-8 consumption of volunteer B | R5-2-1 | Strain1 isolated at day 5 after P-8 consumption of Rat5-2 |
| B6-1 | Strain1 isolated at day 6 after P-8 consumption of volunteer B | R5-2-2 | Strain2 isolated at day 5 after P-8 consumption of Rat5-2 |
| B6-2 | Strain2 isolated at day 6 after P-8 consumption of volunteer B | R5-3-1 | Strain1 isolated at day 5 after P-8 consumption of Rat5-3 |
| B7-1 | Strain1 isolated at day 7 after P-8 consumption of volunteer B | R5-3-2 | Strain2 isolated at day 5 after P-8 consumption of Rat5-3 |
| B7-2 | Strain2 isolated at day 7 after P-8 consumption of volunteer B | R6-1-1 | Strain1 isolated at day 6 after P-8 consumption of Rat6-1 |
| B14-1 | Strain1 isolated at day 14 after P-8 consumption of volunteer B | R6-1-2 | Strain2 isolated at day 6 after P-8 consumption of Rat6-1 |
| B14-2 | Strain2 isolated at day 14 after P-8 consumption of volunteer B | R6-2-1 | Strain1 isolated at day 6 after P-8 consumption of Rat6-2 |
| C1-1 | Strain1 isolated at day 1 after P-8 consumption of volunteer C | R6-2-2 | Strain2 isolated at day 6 after P-8 consumption of Rat6-2 |
| C1-2 | Strain2 isolated at day 1 after P-8 consumption of volunteer C | R6-3-1 | Strain1 isolated at day 6 after P-8 consumption of Rat6-3 |
| C2-1 | Strain1 isolated at day 2 after P-8 consumption of volunteer C | R6-3-2 | Strain2 isolated at day 6 after P-8 consumption of Rat6-3 |
| C2-2 | Strain2 isolated at day 2 after P-8 consumption of volunteer C | R7-1-1 | Strain1 isolated at day 7 after P-8 consumption of Rat7-1 |
| C3-1 | Strain1 isolated at day 3 after P-8 consumption of volunteer C | R7-1-2 | Strain2 isolated at day 7 after P-8 consumption of Rat7-1 |
| C3-2 | Strain2 isolated at day 3 after P-8 consumption of volunteer C | R7-2-1 | Strain1 isolated at day 7 after P-8 consumption of Rat7-2 |
| C4-1 | Strain1 isolated at day 4 after P-8 consumption of volunteer C | R7-2-2 | Strain2 isolated at day 7 after P-8 consumption of Rat7-2 |
| C4-2 | Strain2 isolated at day 4 after P-8 consumption of volunteer C | R14-1-1 | Strain1 isolated at day 14 after P-8 consumption of Rat14-1 |
| C5-1 | Strain1 isolated at day 5 after P-8 consumption of volunteer C | R14-1-2 | Strain2 isolated at day 14 after P-8 consumption of Rat14-1 |
| C5-2 | Strain2 isolated at day 5 after P-8 consumption of volunteer C | R14-2-1 | Strain1 isolated at day 14 after P-8 consumption of Rat14-2 |
| C6-1 | Strain1 isolated at day 6 after P-8 consumption of volunteer C | R14-2-2 | Strain2 isolated at day 14 after P-8 consumption of Rat14-2 |
| C6-2 | Strain2 isolated at day 6 after P-8 consumption of volunteer C | R14-3-1 | Strain1 isolated at day 14 after P-8 consumption of Rat14-3 |
| C7-1 | Strain1 isolated at day 7 after P-8 consumption of volunteer C | R14-3-2 | Strain2 isolated at day 14 after P-8 consumption of Rat14-3 |
| C7-2 | Strain2 isolated at day 7 after P-8 consumption of volunteer C | M4-1 | The 1st week after P-8 consumption period of volunteer M4 |
| C14-1 | Strain1 isolated at day 14 after P-8 consumption of volunteer C | M4-2 | The 2nd week after P-8 consumption period of volunteer M4 |
| C14-2 | Strain2 isolated at day 14 after P-8 consumption of volunteer C | M4-3 | The 4rd week after P-8 consumption period of volunteer M4 |
| E6-1 | The 1st week after P-8 consumption period of volunteer E6 | M4-4 | The 9th week after P-8 consumption period of volunteer M4 |
| E6-2 | The 2nd week after P-8 consumption period of volunteer E6 | Y2-1 | The 1st week after P-8 consumption period of volunteer Y2 |
| E6-3 | The 4rd week after P-8 consumption period of volunteer E6 | Y2-2 | The 2nd week after P-8 consumption period of volunteer Y2 |
| E6-4 | The 9th week after P-8 consumption period of volunteer E6 | Y2-3 | The 4rd week after P-8 consumption period of volunteer Y2 |
| E6-5 | The 13th week after P-8 consumption period of volunteer E6 | Y2-4 | The 9th week after P-8 consumption period of volunteer Y2 |
| E6-6 | The 17th week after P-8 consumption period of volunteer E6 | Y2-5 | The 13th week after P-8 consumption period of volunteer Y2 |

| Table S3. The informations of primers used for SNP validation. | | | | | | | |
| --- | --- | --- | --- | --- | --- | --- | --- |
| Primer Name | Seq(5'-3') | Primer_len | Primer_Pos on frag | frag_pos on genome | GC% | Tm℃ | Target_len |
| SNP02.primerF | TAGTTATGCCGCTTCAATG | 19 | 55 | 710532-711132 | 42.1 | 52.3 | 341 |
| SNP02.primerR | AGGCGAATAGGCTGGGAC | 18 | 453 |  | 61.1 | 57.3 |  |
| SNP05.primerF | AAAATCTCCGTGAACTCC | 18 | 123 | 2188236-2188836 | 44.4 | 48.9 | 347 |
| SNP05.primerR | AATTGCCGTGATAGGC | 16 | 469 |  | 50 | 48.4 |  |
| SNP06.primerF | GGAGCCGTAGTCGTTT | 16 | 145 | 2228159-2228759 | 56.3 | 47.4 | 221 |
| SNP06.primerR | GTTCGGTGATTTCTTT | 16 | 365 |  | 37.5 | 40.8 |  |
| SNP07.primerF | ATGGCGTCATTTGTTCC | 17 | 173 | 2252867-2253467 | 47.1 | 51 | 372 |
| SNP07.primerR | CGTCAGCCGATTGTCC | 16 | 544 |  | 62.5 | 51.9 |  |
| SNP09.primerF | TAGCAACCGGGCAACTGA | 18 | 179 | 2570386-2570986 | 55.6 | 57.9 | 299 |
| SNP09.primerR | AATGGCAAATCGCACGAC | 18 | 477 |  | 50 | 57.2 |  |
| SNP16.primerF | TACGGACCAAGGATG | 15 | 191 | 1374980-1375580 | 53.3 | 42.2 | 337 |
| SNP16.primerR | GGGACGACGATTACA | 15 | 527 |  | 53.3 | 42 |  |
| SNP17.primerF | GCAATGCCTTGACTGG | 16 | 194 | 1058918-1059518 | 56.3 | 49.1 | 343 |
| SNP17.primerR | CTTGGCGAGGTAGACG | 16 | 536 |  | 62.5 | 48.8 |  |
| Note: Several SNP sites were selected to be validated. | | |  |  |  |  |  |

| Table S4. The information of the Plasmid-specific primers used in this study. | | | | | | | |
| --- | --- | --- | --- | --- | --- | --- | --- |
| Name | Sequence (5'→3') | Size (bp) | Start_Pos | Stop_pos | Tm (℃) | GC content (%) | Len_product (bp) |
| LBP1_F1 | GCTCGGTTATGCCAGAAAGA | 20 | 35946 | 35965 | 58 | 50 | 991 |
| LBP1_R1 | ATCAATTCCCGTCGTTCAGC | 20 | 36936 | 36917 | 58.6 | 50 |  |
| LBP2_F1 | TCTTTGGGTTTTGGCAGACC | 20 | 32110 | 32129 | 58.6 | 50 | 972 |
| LBP2_R1 | ACTTAAACCAAGCAGTAATCTTTTT | 25 | 33081 | 33057 | 55.9 | 28 |  |
| LBP3_F1 | CGTCAGTGAAGCCGGAATTA | 20 | 27447 | 27466 | 58 | 50 | 317 |
| LBP3_R1 | TGCACCAGATCCTGTAAAACT | 21 | 27763 | 27743 | 57.2 | 42.9 |  |
| LBP4_F1 | GGTGCCACCCTAGATGTGTT | 20 | 33368 | 33387 | 59.7 | 55 | 959 |
| LBP4_R1 | TGGTGAAATGCCTCTTAGAACCT | 23 | 34326 | 34304 | 59.7 | 43.5 |  |
| LBP5_F1 | TAAACCGGGGGAACACAGTA | 20 | 14513 | 14532 | 58.3 | 50 | 630 |
| LBP5_R1 | CTCCCCCTAATCCCCCTAAT | 20 | 15142 | 15123 | 57.2 | 55 |  |
| LBP6_F1 | AAAAAGTGGTGGCGTTTTGG | 20 | 7731 | 7750 | 58 | 45 | 647 |
| LBP6_R1 | GATGGCAGTCCTTACTCCAC | 20 | 8377 | 8358 | 57.7 | 55 |  |
| LBP7_F1 | AGCAGGAGATGGCGTTTTAG | 20 | 10699 | 10718 | 58 | 50 | 437 |
| LBP7_R1 | AGCACCGCATTCACTTAACA | 20 | 11135 | 11116 | 58.1 | 45 |  |
| Note: Tm represents the annealing temperature | | | | | | | |

| Table S5. The information of all P-8 re-isolates genomes. | | | | | | | | | | |
| --- | --- | --- | --- | --- | --- | --- | --- | --- | --- | --- |
| Samples | No. of scaf | Length(Mb) | Gap(bp) | AverageLength(bp) | N50(bp) | N90(bp) | MaxLength(bp) | MinLength(bp) | GC Content(%) | Accession No. |
| E6-1 | 106 | 3.08 | 1880 | 29056.2 | 92492 | 26102 | 251470 | 211 | 44.63 | MSTY00000000 |
| E6-2 | 107 | 3.08 | 1325 | 28765.82 | 99663 | 26147 | 251509 | 201 | 44.63 | MSTZ00000000 |
| E6-3 | 107 | 3.08 | 1338 | 28778.48 | 99663 | 23039 | 251488 | 208 | 44.63 | MSUA00000000 |
| E6-4 | 105 | 3.08 | 767 | 29316.38 | 92492 | 23039 | 251596 | 205 | 44.63 | MSUB00000000 |
| E6-5 | 109 | 3.08 | 1142 | 28256.35 | 99663 | 23039 | 251530 | 200 | 44.63 | MSUC00000000 |
| E6-6 | 105 | 3.08 | 1638 | 29322.45 | 93882 | 23039 | 251610 | 204 | 44.63 | MSUD00000000 |
| M4-1 | 160 | 3.12 | 2603 | 19492.18 | 86844 | 20155 | 251887 | 200 | 44.64 | MSUE00000000 |
| M4-2 | 128 | 3.11 | 1749 | 24311.17 | 86889 | 20331 | 251562 | 200 | 44.62 | MSUF00000000 |
| M4-3 | 145 | 3.12 | 1957 | 21490.66 | 86844 | 20331 | 251558 | 206 | 44.62 | MSUG00000000 |
| M4-4 | 157 | 3.12 | 1987 | 19891.48 | 85200 | 20331 | 251562 | 200 | 44.59 | MSUH00000000 |
| Y2-1 | 269 | 3.06 | 1227 | 11282.57 | 53463 | 8001 | 174593 | 200 | 44.85 | MSUI00000000 |
| Y2-2 | 209 | 3.12 | 8704 | 14904.58 | 85200 | 20237 | 252203 | 200 | 44.6 | MSUJ00000000 |
| Y2-3 | 141 | 3.08 | 4984 | 21858.58 | 86844 | 22662 | 252218 | 200 | 44.63 | MSUK00000000 |
| Y2-4 | 185 | 3.09 | 26002 | 16698.15 | 68289 | 13219 | 238443 | 204 | 44.65 | MSUL00000000 |
| Y2-5 | 108 | 3.08 | 934 | 28500.31 | 92570 | 20315 | 229102 | 200 | 44.63 | MSUM00000000 |
| R1-1-1 | 134 | 3.14 | 29383 | 23433.27 | 83998 | 19887 | 231029 | 200 | 44.6 | MSVT00000000 |
| R1-1-2 | 137 | 3.15 | 36256 | 22960.62 | 65546 | 17380 | 194903 | 200 | 44.6 | MSVU00000000 |
| R1-2-1 | 161 | 3.18 | 51834 | 19779.05 | 65491 | 15318 | 151722 | 200 | 44.56 | MSVV00000000 |
| R1-2-2 | 185 | 3.15 | 47684 | 17609.8 | 53889 | 15377 | 118958 | 200 | 44.64 | MSVW00000000 |
| R1-3-1 | 152 | 3.18 | 39219 | 20905.83 | 75252 | 17285 | 231437 | 200 | 44.57 | MSVX00000000 |
| R1-3-2 | 162 | 3.19 | 37297 | 19666.88 | 76113 | 16573 | 251552 | 200 | 44.56 | MSVY00000000 |
| R2-1-1 | 139 | 3.17 | 31918 | 22838.37 | 85440 | 19729 | 253338 | 200 | 44.56 | MSVZ00000000 |
| R2-1-2 | 145 | 3.17 | 35367 | 21895.52 | 85618 | 17194 | 231136 | 200 | 44.57 | MSWA00000000 |
| R2-2-1 | 142 | 3.17 | 33148 | 22321.87 | 85651 | 16998 | 212063 | 207 | 44.56 | MSWB00000000 |
| R2-2-2 | 136 | 3.17 | 32497 | 23289.87 | 84870 | 19705 | 230758 | 200 | 44.56 | MSWC00000000 |
| R2-3-1 | 121 | 3.14 | 30180 | 25979.71 | 85208 | 19729 | 253495 | 200 | 44.61 | MSWD00000000 |
| R2-3-2 | 150 | 3.18 | 32136 | 21185.65 | 69007 | 16878 | 253137 | 200 | 44.55 | MSWE00000000 |
| R3-1-1 | 142 | 3.17 | 31342 | 22326.93 | 76245 | 19717 | 230743 | 200 | 44.56 | MSWF00000000 |
| R3-1-2 | 148 | 3.17 | 37014 | 21447.28 | 72441 | 17169 | 194014 | 200 | 44.56 | MSWG00000000 |
| R3-2-1 | 121 | 3.14 | 28786 | 25953.63 | 87045 | 19996 | 231694 | 200 | 44.61 | MSWH00000000 |
| R3-2-2 | 121 | 3.14 | 28822 | 25951.63 | 84731 | 19913 | 194001 | 200 | 44.61 | MSWI00000000 |
| R3-3-1 | 133 | 3.14 | 25788 | 23574.23 | 76059 | 19830 | 230365 | 200 | 44.6 | MSWJ00000000 |
| R3-3-2 | 161 | 3.18 | 30229 | 19749.29 | 84743 | 19729 | 253073 | 200 | 44.55 | MSWK00000000 |
| R4-1-1 | 161 | 3.17 | 34479 | 19709.39 | 65309 | 15329 | 155344 | 200 | 44.56 | MSWL00000000 |
| R4-2-1 | 139 | 3.18 | 30772 | 22877.4 | 76539 | 19907 | 212375 | 200 | 44.57 | MSWM00000000 |
| R4-2-2 | 131 | 3.15 | 36930 | 24037.05 | 76093 | 19705 | 193960 | 200 | 44.57 | MSWN00000000 |
| R4-3-1 | 143 | 3.17 | 34011 | 22196.9 | 85406 | 19717 | 230875 | 200 | 44.56 | MSWO00000000 |
| R4-3-2 | 146 | 3.18 | 37272 | 21759.71 | 76115 | 19717 | 194000 | 200 | 44.56 | MSWP00000000 |
| R5-1-1 | 194 | 3.19 | 37174 | 16463.69 | 79007 | 15341 | 194336 | 200 | 44.55 | MSWQ00000000 |
| R5-1-2 | 118 | 3.13 | 23913 | 26553.84 | 87004 | 20144 | 230699 | 200 | 44.6 | MSWR00000000 |
| R5-2-1 | 152 | 3.17 | 35171 | 20882.34 | 65234 | 19717 | 193862 | 200 | 44.57 | MSWS00000000 |
| R5-2-2 | 143 | 3.18 | 36506 | 22206.78 | 80068 | 19717 | 155339 | 200 | 44.56 | MSWT00000000 |
| R5-3-1 | 155 | 3.18 | 34340 | 20498.14 | 85122 | 19717 | 230759 | 200 | 44.56 | MSWU00000000 |
| R5-3-2 | 143 | 3.17 | 32117 | 22180.67 | 79450 | 19717 | 155346 | 204 | 44.56 | MSWV00000000 |
| R6-1-1 | 141 | 3.18 | 36193 | 22524.08 | 76098 | 19717 | 232030 | 200 | 44.56 | MSWW00000000 |
| R6-1-2 | 143 | 3.17 | 35890 | 22197.73 | 85632 | 19717 | 231489 | 200 | 44.56 | MSWX00000000 |
| R6-2-1 | 119 | 3.11 | 22927 | 26111.29 | 94001 | 23317 | 251658 | 200 | 44.64 | MSWY00000000 |
| R6-2-2 | 115 | 3.10 | 18002 | 26990.15 | 76306 | 23249 | 253289 | 200 | 44.65 | MSWZ00000000 |
| R6-3-1 | 146 | 3.17 | 33351 | 21727.64 | 76101 | 17635 | 230729 | 200 | 44.56 | MSXA00000000 |
| R6-3-2 | 136 | 3.17 | 25869 | 23283.45 | 84869 | 19717 | 230414 | 200 | 44.55 | MSXB00000000 |
| R7-1-1 | 144 | 3.18 | 39577 | 22054.4 | 85154 | 19705 | 231949 | 200 | 44.57 | MSXC00000000 |
| R7-1-2 | 132 | 3.16 | 26106 | 23940.53 | 92964 | 23182 | 230285 | 200 | 44.56 | MSXD00000000 |
| R7-2-1 | 121 | 3.14 | 31014 | 25957.74 | 77704 | 19717 | 155345 | 200 | 44.61 | MSXE00000000 |
| R7-2-2 | 163 | 3.15 | 42589 | 19313.13 | 50947 | 16487 | 102496 | 200 | 44.61 | MSXF00000000 |
| R14-1-1 | 146 | 3.17 | 32048 | 21732.4 | 76108 | 17085 | 155337 | 200 | 44.56 | MSXG00000000 |
| R14-1-2 | 134 | 3.17 | 27346 | 23636.9 | 96753 | 19913 | 230555 | 200 | 44.56 | MSXH00000000 |
| R14-2-1 | 134 | 3.14 | 30561 | 23421.09 | 72828 | 20041 | 155355 | 202 | 44.6 | MSXI00000000 |
| R14-2-2 | 123 | 3.13 | 20690 | 25463.69 | 86972 | 20144 | 230084 | 200 | 44.59 | MSXJ00000000 |
| R14-3-1 | 126 | 3.13 | 22709 | 24867.56 | 85498 | 19996 | 230503 | 200 | 44.59 | MSXK00000000 |
| R14-3-2 | 133 | 3.14 | 28113 | 23596.15 | 85668 | 20053 | 155348 | 200 | 44.59 | MSXL00000000 |
| Average | 141.87 | 3.14 | 25380.12 | 22816.31 | 81104.92 | 19446.62 | 217725.17 | 200.87 | 44.59 |  |
| Std | 27.67 | 0.04 | 14058.49 | 3577.28 | 10746.31 | 2905.79 | 38347.88 | 2.27 | 0.05 |  |

| Table. S6 The funtional annotation information for 4 lost plasmids. | | | | | | | |
| --- | --- | --- | --- | --- | --- | --- | --- |
|  | Gene_ID | COG_ID | COG_class | COG_function | COG_categories | KEGG | Pathway |
| LBPp1 | LBP_RS14510 | COG1113 | E | Gamma-aminobutyrate permease and related permeases | Amino acid transport and metabolism | K03293 |  |
| LBPp1 | LBP_RS14365 | COG4975 | G | Putative glucose uptake permease | Carbohydrate transport and metabolism | K05340 |  |
| LBPp1 | LBP_RS14375 | COG0662 | G | Mannose-6-phosphate isomerase | Carbohydrate transport and metabolism |  |  |
| LBPp1 | LBP_RS14565 | COG2211 | G | Na+/melibiose symporter and related transporters | Carbohydrate transport and metabolism | K16209 |  |
| LBPp1 | LBP_RS14435 | COG1321 | K | Mn-dependent transcriptional regulator | Transcription | K03709 |  |
| LBPp1 | LBP_RS14420 | COG1961 | L | Site-specific recombinases, DNA invertase Pin homologs | Replication, recombination and repair |  |  |
| LBPp1 | LBP_RS14410 | COG2963 | L | Transposase and inactivated derivatives | Replication, recombination and repair |  |  |
| LBPp1 | LBP_RS14430 | COG1914 | P | Mn2+ and Fe2+ transporters of the NRAMP family | Inorganic ion transport and metabolism | K03322 |  |
| LBPp1 | LBP_RS14440 | COG0861 | P | Membrane protein TerC, possibly involved in tellurium resistance | Inorganic ion transport and metabolism |  |  |
| LBPp1 | LBP_RS14340 | COG1418 | R | Predicted HD superfamily hydrolase | General function prediction only | K06950 |  |
| LBPp1 | LBP_RS14570 | COG3560 | R | Predicted oxidoreductase related to nitroreductase | General function prediction only | K07078 |  |
| LBPp2 | LBP_RS14790 | COG1249 | C | Pyruvate/2-oxoglutarate dehydrogenase complex, dihydrolipoamide dehydrogenase (E3) component, and related enzymes | Energy production and conversion |  |  |
| LBPp2 | LBP_RS14755 | COG1192 | D | ATPases involved in chromosome partitioning | Cell cycle control, cell division, chromosome partitioning | K03496 |  |
| LBPp2 | LBP_RS14600 | COG3250 | G | Beta-galactosidase/beta-glucuronidase | Carbohydrate transport and metabolism | K01190 | Metabolism;Carbohydrate metabolism;Galactose metabolism [PATH:ko00052]\|Metabolism;Lipid metabolism;Sphingolipid metabolism [PATH:ko00600]\|Metabolism;Glycan biosynthesis and metabolism;Other glycan degradation [PATH:ko00511] |
| LBPp2 | LBP_RS14595 | COG3250 | G | Beta-galactosidase/beta-glucuronidase | Carbohydrate transport and metabolism | K01190 | Metabolism;Carbohydrate metabolism;Galactose metabolism [PATH:ko00052]\|Metabolism;Lipid metabolism;Sphingolipid metabolism [PATH:ko00600]\|Metabolism;Glycan biosynthesis and metabolism;Other glycan degradation [PATH:ko00511] |
| LBPp2 | LBP_RS14590 | COG3345 | G | Alpha-galactosidase | Carbohydrate transport and metabolism | K07407 | Metabolism;Carbohydrate metabolism;Galactose metabolism [PATH:ko00052]\|Metabolism;Lipid metabolism;Glycerolipid metabolism [PATH:ko00561]\|Metabolism;Lipid metabolism;Sphingolipid metabolism [PATH:ko00600]\|Metabolism;Glycan biosynthesis and metabolism;Glycosphingolipid biosynthesis - globo series [PATH:ko00603] |
| LBPp2 | LBP_RS14615 | COG1874 | G | Beta-galactosidase | Carbohydrate transport and metabolism | K12308 | Metabolism;Carbohydrate metabolism;Galactose metabolism [PATH:ko00052] |
| LBPp2 | LBP_RS14620 | COG2211 | G | Na+/melibiose symporter and related transporters | Carbohydrate transport and metabolism | K16209 |  |
| LBPp2 | LBP_RS14740 | COG1321 | K | Mn-dependent transcriptional regulator | Transcription | K03709 |  |
| LBPp2 | LBP_RS14745 | COG3316 | L | Transposase and inactivated derivatives | Replication, recombination and repair | K07498 |  |
| LBPp2 | LBP_RS14725 | COG1961 | L | Site-specific recombinases, DNA invertase Pin homologs | Replication, recombination and repair |  |  |
| LBPp2 | LBP_RS14680 | COG3328 | L | Transposase and inactivated derivatives | Replication, recombination and repair |  |  |
| LBPp2 | LBP_RS14735 | COG1914 | P | Mn2+ and Fe2+ transporters of the NRAMP family | Inorganic ion transport and metabolism | K03322 |  |
| LBPp2 | LBP_RS14715 | COG3158 | P | K+ transporter | Inorganic ion transport and metabolism | K03549 |  |
| LBPp2 | LBP_RS14710 | COG0745 | TK | Response regulators consisting of a CheY-like receiver domain and a winged-helix DNA-binding domain | Signal transduction mechanisms; Transcription | K07667 | Environmental Information Processing;Signal transduction;Two-component system [PATH:ko02020] |
| LBPp4 | LBP_RS15130 | COG1192 | D | ATPases involved in chromosome partitioning | Cell cycle control, cell division, chromosome partitioning | K03496 |  |
| LBPp4 | LBP_RS15110 | COG2376 | G | Dihydroxyacetone kinase | Carbohydrate transport and metabolism | K05878 | Metabolism;Lipid metabolism;Glycerolipid metabolism [PATH:ko00561] |
| LBPp4 | LBP_RS15105 | COG2376 | G | Dihydroxyacetone kinase | Carbohydrate transport and metabolism | K05879 | Metabolism;Lipid metabolism;Glycerolipid metabolism [PATH:ko00561] |
| LBPp4 | LBP_RS15045 | COG0477 | GEPR | Permeases of the major facilitator superfamily | Carbohydrate transport and metabolism;Amino acid transport and metabolism;Inorganic ion transport and metabolism;General function prediction only | K08217 |  |
| LBPp4 | LBP_RS15205 | COG1961 | L | Site-specific recombinases, DNA invertase Pin homologs | Replication, recombination and repair |  |  |
| LBPp4 | LBP_RS15070 | COG2826 | L | Transposase and inactivated derivatives, IS30 family | Replication, recombination and repair |  |  |
| LBPp4 | LBP_RS15075 | COG3328 | L | Transposase and inactivated derivatives | Replication, recombination and repair |  |  |
| LBPp4 | LBP_RS15165 | COG0550 | L | Topoisomerase IA | Replication, recombination and repair | K03169 |  |
| LBPp4 | LBP_RS16520 | COG2189 | L | Adenine specific DNA methylase Mod | Replication, recombination and repair | K07316 |  |
| LBPp4 | LBP_RS15040 | COG2217 | P | Cation transport ATPase | Inorganic ion transport and metabolism | K01533 |  |
| LBPp4 | LBP_RS15090 | COG4716 | S | Myosin-crossreactive antigen | Function unknown | K10254 |  |
| LBPp4 | LBP_RS15100 | COG3412 | S | Uncharacterized protein conserved in bacteria | Function unknown |  |  |
| LBPp4 | LBP_RS15185 | COG0286 | V | Type I restriction-modification system methyltransferase subunit | Defense mechanisms | K03427 |  |
| LBPp4 | LBP_RS15190 | COG0610 | V | Type I site-specific restriction-modification system, R (restriction) subunit and related helicases | Defense mechanisms | K01153 |  |
| LBPp4 | LBP_RS16560 | COG0732 | V | Restriction endonuclease S subunits | Defense mechanisms | K01154 |  |
| LBPp7 | LBP_RS15370 | COG1126 | E | ABC-type polar amino acid transport system, ATPase component | Amino acid transport and metabolism | K02028 |  |
| LBPp7 | LBP_RS15360 | COG0765 | E | ABC-type amino acid transport system, permease component | Amino acid transport and metabolism | K02029 |  |
| LBPp7 | LBP_RS15365 | COG0765 | E | ABC-type amino acid transport system, permease component | Amino acid transport and metabolism | K02029 |  |
| LBPp7 | LBP_RS15375 | COG0834 | ET | ABC-type amino acid transport/signal transduction systems, periplasmic component/domain | Amino acid transport and metabolism; Signal transduction mechanisms | K02030 |  |
| LBPp7 | LBP_RS15435 | COG1575 | H | 1,4-dihydroxy-2-naphthoate octaprenyltransferase | Coenzyme transport and metabolism | K02548 | Metabolism;Metabolism of cofactors and vitamins;Ubiquinone and other terpenoid-quinone biosynthesis [PATH:ko00130] |
| LBPp7 | LBP_RS15420 | COG1278 | K | Cold shock proteins | Transcription | K03704 |  |
| LBPp7 | LBP_RS15400 | COG0582 | L | Integrase | Replication, recombination and repair |  |  |
| LBPp7 | LBP_RS15440 | COG3293 | L | Transposase and inactivated derivatives | Replication, recombination and repair |  |  |
| LBPp7 | LBP_RS15390 | COG2337 | T | Growth inhibitor | Signal transduction mechanisms | K07171 |  |
